# Neural Activations during Visual Sequence Learning Leave a Trace in Post-Training Spontaneous EEG

**DOI:** 10.1371/journal.pone.0065882

**Published:** 2013-06-14

**Authors:** Clara Moisello, Hadj Boumediene Meziane, Simon Kelly, Bernardo Perfetti, Svetlana Kvint, Nicholas Voutsinas, Daniella Blanco, Angelo Quartarone, Giulio Tononi, Maria Felice Ghilardi

**Affiliations:** 1 Department of Physiology, Pharmacology and Neuroscience, City University of New York Medical School, New York, New York, United States of America; 2 Department of Biomedical Engineering, City College of New York, New York, New York, United States of America; 3 Department of Neurosciences, Psychiatry and Anaesthesiological Science, University of Messina, Messina, Italy; 4 Department of Psychiatry, University of Wisconsin, Madison, Wisconsin, United States of America; Hangzhou Normal University, China

## Abstract

Recent EEG studies have shown that implicit learning involving specific cortical circuits results in an enduring local trace manifested as local changes in spectral power. Here we used a well characterized visual sequence learning task and high density-(hd-)EEG recording to determine whether also declarative learning leaves a post-task, local change in the resting state oscillatory activity in the areas involved in the learning process. Thus, we recorded hd-EEG in normal subjects before, during and after the acquisition of the order of a fixed spatial target sequence (VSEQ) and during the presentation of targets in random order (VRAN). We first determined the temporal evolution of spectral changes during VSEQ and compared it to VRAN. We found significant differences in the alpha and theta bands in three main scalp regions, a right occipito-parietal (ROP), an anterior-frontal (AFr), and a right frontal (RFr) area. The changes in frontal theta power during VSEQ were positively correlated with the learning rate. Further, post-learning EEG recordings during resting state revealed a significant increase in alpha power in ROP relative to a pre-learning baseline. We conclude that declarative learning is associated with alpha and theta changes in frontal and posterior regions that occur during the task, and with an increase of alpha power in the occipito-parietal region after the task. These post-task changes may represent a trace of learning and a hallmark of use-dependent plasticity.

## Introduction

Animal studies have shown that acquisition of new material or novel experiences modifies the synaptic landscape in the brain regions involved in the tasks through synaptic potentiation processes [1], [2]. A few studies in humans have demonstrated that learning or repetitive activity leave local traces that can be detected, immediately after the performance, with imaging and stimulation techniques [3], [4], [5]. The notion that post-performance traces are local and task-specific has been confirmed by recent studies. In particular, Hung and co-workers [6], [7] have shown task-specific traces in the spontaneous EEG after twenty-four hours of continuous performance: theta increases were found in parieto-occipital areas after a driving video game, and over language-related areas after listening to audio-books. Similar traces in the spontaneous EEG could also be detected after tasks of shorter duration: after a forty-minute motor task where subjects implicitly learned to adapt their movements to a rotated display [8], [9], [10], we found significant changes in alpha power in resting-state EEG [7] in regions that, in previous studies, showed EEG changes during the task [11]. Altogether, these results suggest that task performance leads to post-task changes in the spontaneous resting-state EEG, which are local and specific to the areas involved in the task. The main scope of the present work is to confirm the occurrence of local changes after the performance of a forty-minute task with declarative spatial learning attributes. To this aim, we examined the changes in spontaneous EEG following forty-minute training on a sequence-learning task that emphasizes the declarative aspects of order acquisition. While imaging studies with O^15^-PET [9], [12], [13] have consistently shown that frontal and parieto-occipital regions are especially involved in the acquisition of spatial sequences with this learning task, there are no EEG studies characterizing the fine temporal dynamics of the processes involved in this type of sequence learning over the entire scalp. Thus, as a first step to determine whether the post-task changes in the spontaneous resting-state EEG occurred in the areas involved in the task, we initially defined the topography and the time course of oscillatory activity changes of sequence learning compared to a non-learning control task with similar visual characteristics. We focused on theta and alpha bands because in cognitive and semantic memory formation studies, theta power increase (i.e., synchronization) has been associated with the encoding of new information [14], [15], [16], whereas alpha power decrease (i.e., desynchronization) has been linked with the activation of memory traces and access to memory storage [17], [18], [19]. We then compared the spontaneous EEG recordings during a period of resting state before and after the sequence learning. We found that the learning of a spatial sequence order, a type of declarative learning, is accompanied by changes of oscillatory activity in electrodes over frontal and posterior regions and, most importantly, leaves a post-task trace in the spontaneous EEG detected in electrodes over areas involved during the task.

## Materials and Methods

### Subjects

Twenty-one subjects (mean age ± SD: 24.2±4.8 years; 13 men) participated in the study. They were all right handed as determined by the Edinburgh inventory [20], had normal or corrected vision and no history of neurological or psychiatric disorders.

### Ethics Statement

The experiments were conducted with the approval of the Institutional Review Boards of the participating institutions according to the principles expressed in the Declaration of Helsinki. All participants signed a written informed consent form and were naïve to the purpose of the study.

### Experimental Setup, Design and Tasks

**Figure 1:**
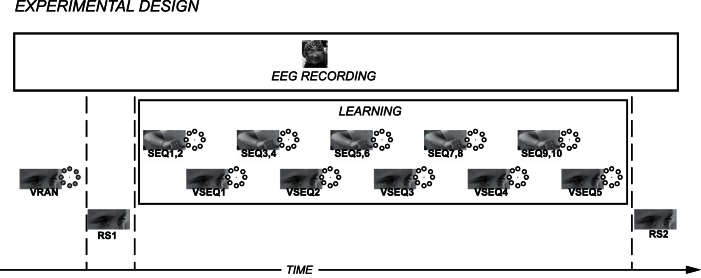
Experimental design for sequence learning. First, subjects performed three blocks of VRAN, a control task for VSEQ, where targets appeared randomly and instructions were to maintain attention avoiding eye movements. Then, three-minute resting state EEG was recorded (RS1), followed by alternating blocks of visuo-motor (SEQ) and visual (VSEQ) sequence learning tasks. At the end of the session, three-minute resting state EEG was recorded (RS2). The eight-target array is represented nearby each block. Please note that the subject of the photograph has given written informed consent, as outlined in the PLOS consent form, to publication of their photograph.

Subjects were seated in front of a computer screen and were outfitted with a 256-electrode cap for hd-EEG recordings. As illustrated in Figure 1, hd-EEG was recorded during the entire experiment, which included two three-minute recordings of resting state and three tasks. During the resting state, subjects were asked to relax, to keep their eyes open and to fixate on a point that was constantly present in the center of the screen. The three tasks performed during the experiment, VRAN, VSEQ and SEQ, have been described in detail previously [21], [22], [23] and in the following paragraphs. Figure 1 illustrates the study design. First, subjects performed three blocks of VRAN, a control task for VSEQ, where one of eight targets appeared for 200 ms on a screen in a non-repeating and unpredictable order (48 targets per block, presentation rate 1.5 s). Targets were eight radially-arrayed circles (1 cm diameter) at 2 cm-distance from the center. Instructions were to maintain attention on the target array, avoiding eye movements. Afterwards, three minutes of resting state EEG were recorded (RS1). Subjects were then asked to learn a 16-element sequence in alternating blocks of visuo-motor (SEQ) and visual (VSEQ) tasks (Figure 1A). In both SEQ and VSEQ, the eight targets appeared (200 ms stimulus duration) with the same 16-element repeating order at a fixed time interval of 1.5 s (same target array as in VRAN). As each block consisted of 48 target presentations, there were three complete sequence repetitions or cycles per block. Subjects were informed that a 16-element sequence was to be presented and instructed to learn its order: in the VSEQ blocks, they were asked to learn the sequence order without moving; in the SEQ blocks, instead, they had to reach with the their right dominant hand for the appearing targets and, when confident, to anticipate the target appearance. As shown previously, SEQ has the same declarative learning attributes of VSEQ [21], [23], [24]. They performed a total of five VSEQ blocks and ten SEQ blocks (lasting approximately forty minutes). Finally, we recorded a second resting state EEG of three minutes (RS2). At the end of each VSEQ and SEQ block, subjects reported the sequence order and declarative scores (from 0, no sequence detected, to 16, full sequence order) were computed [21], [23], [24]. Learning indices used for correlational analyses included: the number of blocks with full declarative knowledge of the sequence order (i.e., with verbal score of 16); the mean acquisition rate per block (i.e., average of the difference between consecutive blocks in verbal scores).

With a procedure used in previous work for other tasks [11], [25], [26], the EEG recordings for each 1.5 sec epoch in VSEQ and VRAN were first, aligned based on the latencies of stimulus presentation, then, segmented and analyzed in five 250-ms temporal windows (Figure 1B) to define the brain dynamics related to visual sequence learning (see below). Importantly, VSEQ and VRAN have similar attentional and initial stimulus processing requirements, but they differ because VSEQ requires also learning-related processes. Thus, the difference between VSEQ and VRAN should reflect the brain dynamics related to learning. The results of EEG analyses of the SEQ blocks are not reported in the current paper.

To assess the specificity of the trace left in the resting state EEG, we also recorded resting state EEG before and after a separate forty-minute testing session (*control* RS1 and *control* RS2), wherein subjects performed different motor tasks, which did not involve any declarative or implicit learning. Briefly, in this forty-minute control session, they performed in randomized order, 15 blocks (each block duration: approximately 2 minutes with an average of one-minute inter-block interval) of reaching movements. Nine blocks were in choice-reaction time paradigms, with targets in twelve different directions and four distances; six blocks were in timed-response paradigms to eight spatially and temporally predictable targets. After each block, subjects were debriefed about their performance and feedback was provided. The order of the control and the sequence learning sessions was randomized. We selected this type of control session because it allowed for controlling attention and other processes.

### EEG Recording and Signal Processing

HD-EEG was recorded from 256 electrodes (Hydrocel net, Electrical Geodesics Inc.) while subjects performed the tasks and during the afore-mentioned resting state periods. Data were collected at a sampling rate of 250 Hz using the high impedance amplifier Net Amp 300 and Net Station 4.3 (Electrical Geodesics Inc.). Impedances were kept below 50 kΩ. From the original 256 electrodes, we removed 73 channels located on the cheeks and on the neck. The remaining 183 electrodes were used for further analysis. During the recording, the EEG signal was referenced to the Cz sensor. For analysis, data were re-referenced to the average across the 183 electrodes.

#### Pre-processing

We performed data pre-processing with NetStation 4.3 software (Electrical Geodesics Inc.) and the public license toolbox EEGLAB [27], as previously described [11], [25]. Briefly, the continuous EEG signals were filtered with a pass-band of 0.5 Hz to 80 Hz and a notch filter centered at 60 Hz; channels affected by bad scalp-electrode contact were visually identified and replaced with spherical spline interpolation. EEG was then segmented into 4.5-s epochs from −2 s to 2.5 s relative stimulus onset. This epoch length was selected in order to prevent contamination of the EEG with possible effects of filter edge artifacts that might be associated with some of the analyses. Finally, stereotypical artifacts, such as blinks, eye movements and motion-related signals, were removed by Principal Component Analysis (PCA; [28], [29] and any non-stereotypical artifacts by visual inspection. The identification of components accounting for extra-brain artifactual stereotyped sources was based on a well-established procedure detailed elsewhere [29], [30]. Briefly, we visually inspected the power spectral density, topographical maps and time activations of each estimated component, which have been proven to be highly informative on the nature of the signal accounted by the spatial filter. The components identified as “artifactual” were removed from the raw EEG signal. We further visually inspected the power spectral density of the “corrected” raw EEG signals to rule out the presence of any residual artifacts.

#### Spectral estimates

Spectral estimates at all channels were obtained in each participant on a single trials basis by using Matlab 7 (MathWorks, Natick, MA, USA), and the public license toolbox EEGLAB [27]. Time-frequency representations (TFRs) were computed using Morlet Wavelet transforms. Each 4.5-s trial epoch was portioned into 200 time bins (distance between two consecutive bins was 16 ms). The lowest frequency was set at 4 Hz with 3 cycles, resulting in a window size of 209 sample points (836 ms). At the maximum frequency (45 Hz), the cycles were 30. The distance between two output frequency bins was 0.5 Hz. The TFRs of each channel were normalized by subtracting the average power calculated across all channels for all the specific frequency bins.

Task-related power variations were investigated within four frequency bands (theta: 4–8 Hz; alpha 8–13 Hz; beta 13–25 Hz and gamma 25–45 Hz) and five consecutive temporal windows of 250-ms duration: one pre-stimulus window (−250 ms to stimulus onset) and four consecutive post-stimulus windows (ending at 1000 ms). The choice of 250-ms temporal windows was limited to the statistical analysis and was based on visual inspection of the temporal profiles of power variation.

To examine the topography and the time-course of the spectral changes underlying the acquisition of the sequence, we adopted the following approach. First, we compared group spectral estimates between VRAN and the average of the five VSEQ blocks at all channels with paired sample t-tests. For all selected time intervals and frequencies, we constructed topographic maps of significant VSEQ-VRAN differences using a statistical nonparametric mapping procedure (SnPM; simple-threshold, P_perm_ = 0.05). The resulting t-scores were plotted on the scalp by spherical spline interpolation. To take advantage of the actual data distribution and account for multiple comparisons testing in high density EEG recordings, significance criteria were determined on the basis of a permutation test [31] as detailed in previous papers [7], [11]. No significant effects were observed for beta or gamma. However, as further explained in the Results section, the statistical maps for theta and alpha revealed three clusters of significant electrodes, or Regions of Interest (ROIs) over right parietal, midline frontal and right frontal regions. All subsequent analyses were thus performed only on the theta and alpha frequency bands. Repeated measures ANOVAs were used to assess differences in theta and alpha ROI normalized power between VRAN (*visual control condition*), VSEQ1 (*early learning condition)* and VSEQ5 (*late learning condition*) in the five stimulus-locked temporal windows. Significant effects and interactions were further explored with Bonferroni corrected post hoc tests. Finally, the significant power estimates were used in correlational analyses with indices of declarative learning.

#### Analysis of the resting state EEG

Pre-processing and artifact removal were done with the same procedure described in the previous paragraph. For this analysis, continuous data were segmented into 4 s epochs. The power spectrum for each clean data epoch was computed via the fast-Fourier transform (FFT-Hamming window). For each subject, power at each channel was normalized by subtracting the average power on the scalp for each frequency bin (as described before). Power was then averaged within the theta and alpha bands, thus obtaining a value for each subject in each of the four resting sessions (RS1, RS2, *control* RS1 and *control* RS2).

Paired-sample t-tests were used to compare group spectral estimates at all channels between RS2 and RS1, as well as between *control* RS2 and *control* RS1. The resulting T scores were plotted on the scalp by spherical spline interpolation [11].

## Results

### Learning Rate is Increasing Across Blocks

**Figure 2:**
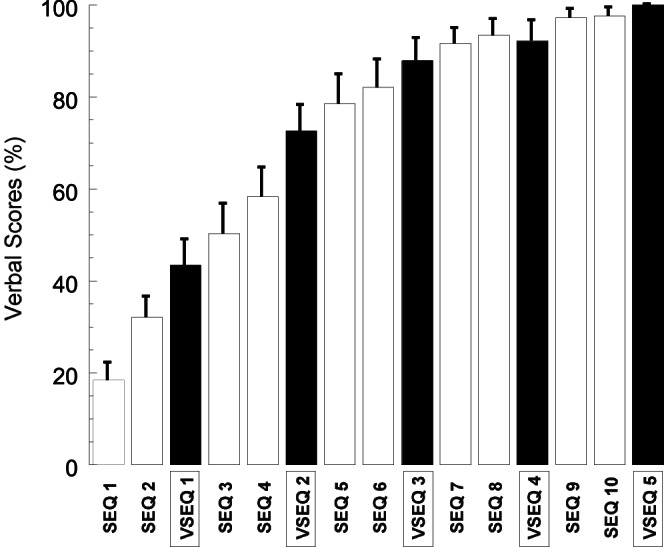
Behavioral results. Mean Verbal Score (±SE) are plotted in percentage per each SEQ (white columns) and VSEQ (black columns) block.

All the subjects learned a 16-element sequence and attained complete knowledge of the sequence by the end of the last VSEQ block. The verbal scores significantly increased across all blocks (Figure 2, repeated measure ANOVA: verbal scores VSEQ+SEQ blocks: F(20, 280) = 69.6; p<0.0001; verbal scores VSEQ blocks only: F(20, 80) = 42.97; p<0.0001). Although all subjects reached the full declarative knowledge of the sequence by the end of the last block, there were wide differences between subjects in the acquisition rate: the number of blocks with full declarative knowledge (i.e., with verbal score of 16) varied from 1 to 13 (mean±SE: 7.71±0.77); the acquisition rate per block (i.e., average of the difference between consecutive blocks in verbal scores) varied from 11.46% to 23.26% (mean±SE: 15.48±0.60%).

### Topography of Spectral Changes during Learning

**Figure 3:**
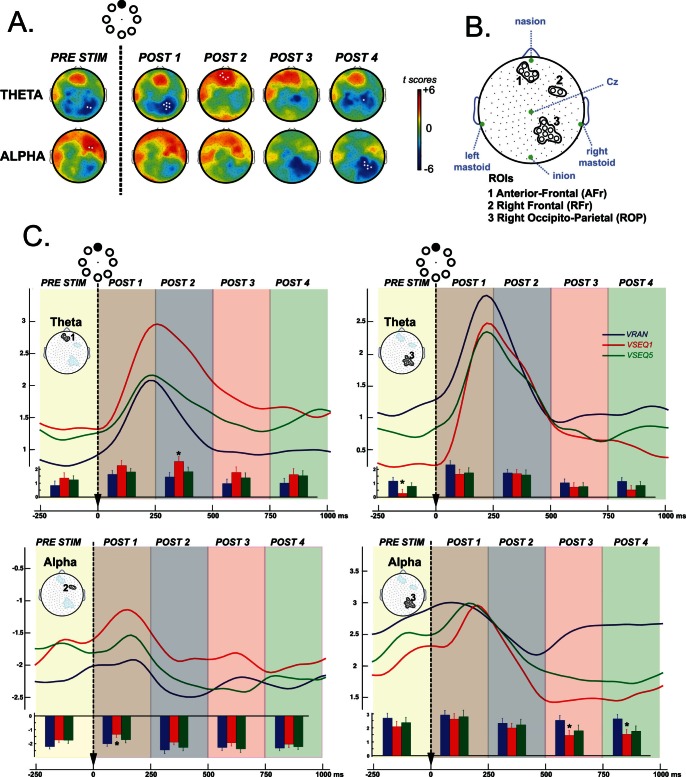
Spectral power analysis. A. Statistical nonparametric mapping analysis of the difference between VRAN and the average of the five blocks of VSEQ in five 250-ms temporal windows for theta and alpha frequency bands. The white circles indicate electrodes with significant power differences. B. Identification of the three ROIs based on significant power differences in A. C. Time course of the average normalized spectral power VRAN (blue), VSEQ1 (red) and VSEQ5 (green) for the three ROIs in B. in theta (upper row) and alpha (lower row) frequency bands. Bars at the bottom of each graph represent the mean power (± SE) for the corresponding time interval, with asterisks indicating significant (p<0.05) contrasts.

We examined the topography and the time-course of the spectral changes underlying the acquisition of the sequence order with power spectral analysis. First, we compared in all frequency bands, the normalized spectral power of the averaged five VSEQ blocks (visual learning) with that of VRAN (control visual condition) using five 250-ms temporal windows (see methods). As shown in Figure 3A–B, such comparison revealed significant differences in theta and alpha bands, which involved electrode sites overlaying three regions, an anterio-frontal (AFr), a right occipito-parietal (ROP) and a right frontal (RFr) area. Figure 3C shows the grand average time course of theta power in the AFr and ROP ROIs, and for the alpha range in the RFr and ROP ROIs in VRAN, VSEQ1 (early learning) and VSEQ5 (late learning). At the end of these two blocks, declarative scores showed the least inter-subject variability.

In the following paragraphs, we describe the temporal changes of theta and alpha for each of the three conditions (VRAN, VSEQ1 and VSEQ5) and their differences.

#### THETA BAND (Figure 3C, upper row)

In both frontal and parietal regions, the changes over time of theta power were similar in the three tasks, with a robust increase after stimulus presentation and a decrease to stable values 500 ms later. Repeated measure ANOVAs (Table 1) showed that in both areas, there was a significant effect of time with a significant interaction between the three tasks and the temporal windows. Although the changes across time were significant in all the tasks (Table 2), there were significant differences between the three tasks for specific temporal windows. Briefly, comparisons between VRAN and VSEQ1 in AFr revealed that theta increase was in general greater in VSEQ1 (Table 3), with significant differences only in the POST2 window (250–500 ms after the stimulus appearance, post hoc test: p = 0.005). Such effects were not present in VSEQ5 (see comparisons VSEQ5 vs. VRAN and VSEQ5 vs. VSEQ1 in Table 3). In ROP, VSEQ1 showed in general, lower levels of theta increase compared to VRAN (Table 3), with a statistical difference in the interval before stimulus appearance (Prestim, post-hoc test: p = 0.001). In VSEQ5, power values on average, returned to the VRAN range (see comparisons VSEQ5 vs. VRAN and VSEQ5 vs. VSEQ1 in Table 3).

**Table 1:** Repeated measure ANOVAs in ROP (right occipito-parietal), AFr (Anterior Frontal) and RF (Right Frontal) areas for Theta and Alpha Bands.

*Effect*	*DF*	*F*	*p*	*power*	*F*	*p*	*power*
		*AFr Theta*			*ROP Theta*		
Condition	2	0.873	0.42	0.19	1.123	0.33	0.23
TemporalBin	4	39.707	**<0.0001**	**1.00**	66.099	**<0.0001**	**1.00**
Cond ×Temp Bin	8	2.707	**0.0073**	**0.94**	2.959	**0.0036**	**0.96**
		***RFr Alpha***			***ROP Alpha***		
Condition	2	2.305	0.11	0.43	1.744	0.18	0.34
TemporalBin	4	10.606	**<0.0001**	**1.00**	17.657	**<0.0001**	**1.00**
Cond ×TempBin	8	2.617	**0.0093**	**0.92**	2.791	**0.0058**	**0.94**

Comparisons were within three tasks or conditions (VRAN, VSEQ1 and VSEQ5) across five temporal bins (PreStim, Post1, Post2, Post3, and Post4). Significant results are highlighted in bold.

**Table 2:** Repeated measures ANOVAs in ROP (right occipito-parietal), AFr (Anterior Frontal) and RF (Right Frontal) areas for Theta and Alpha Bands.

	*DF*	*F*	*P*	*power*	*F*	*p*	*power*
		*AFr Theta*			*ROP Theta*		
VRAN	4	16.977	**<0.0001**	**1.00**	29.872	**<0.0001**	**1.00**
VSEQ1	4	30.662	**<0.0001**	**1.00**	33.148	**<0.0001**	**1.00**
VSEQ5	4	4.872	**<0.0001**	**0.96**	12.261	**<0.0001**	**1.00**
		***RFr Alpha***			***ROP Alpha***		
VRAN	4	2.16	0.0816	0.60	2.01	0.1014	0.57
VSEQ1	4	6.916	**<0.0001**	**1.00**	14.41	**<0.0001**	**1.00**
VSEQ5	4	5.964	**0.0003**	**0.99**	7.69	**<0.0001**	**1.00**

Comparisons were performed across five temporal bins (PreStim, Post1, Post2, Post3, and Post4), separately for VRAN, VSEQ1 and VSEQ5. Significant results are highlighted in bold.

**Table 3:** Repeated measures ANOVAs in ROP (right occipito-parietal), AFr (Anterior Frontal) and RF (Right Frontal) areas for Theta and Alpha Bands.

	*DF*	*F*		*p*	*power*	*F*	*p*	*power*
*VSEQ1 vs. VRAN*		*AFr Theta*				*ROP Theta*		
Condition	1	1.587		0.21	0.22	2.191	0.15	0.29
Temporal Bin	4	44.533		**<0.0001**	**1.00**	58.565	**<0.0001**	**1.00**
Cond ×TempBin	8	4.299		**0.0025**	**0.94**	4.867	**0.001**	**0.96**
*VSEQ1 vs. VSEQ5*								
Condition	1	0.273		0.6	0.08	0.201	0.65	0.07
Temporal Bin	4	25.164		**<0.0001**	**1.00**	40.668	**<0.0001**	**1.00**
Cond ×TempBin	8	3.711		**0.0065**	**0.88**	3.137	**0.0164**	**0.81**
*VSEQ5 vs. VRAN*								
Condition	1	0.72		0.4	0.13	1.076	0.306	0.16
Temporal Bin	4	17.275		**<0.0001**	**1.00**	37.585	**<0.0001**	**1.00**
Cond ×TempBin	8	0.486		0.75	0.16	1.016	0.401	0.31
*VSEQ1 vs. VRAN*		***RFr Alpha***				***ROP Alpha***		
Condition	1	4.893	**0.033**		**0.57**	3.553	0.067	0.44
Temporal Bin	4	6.382	**<0.0001**		**1.00**	10.136	**<0.0001**	**1.00**
Cond ×TempBin	8	3.456	**0.0098**		**0.86**	5.223	**0.0006**	**0.97**
*VSEQ1 vs. VSEQ5*								
Condition	1	2.019	0.164		0.17	0.21	0.65	0.07
Temporal Bin	4	10.127	**<0.0001**		**1.00**	20.752	**<0.0001**	**1.00**
Cond ×TempBin	8	2.799	**0.03**		**0.76**	0.36	0.83	0.13
*VSEQ5 vs. VRAN*								
Condition	1	0.39	0.54		0.09	1.747	0.19	0.25
Temporal Bin	4	7.037	**<0.0001**		**1.00**	7.107	**<0.0001**	**1.00**
Cond ×TempBin	8	1.518	0.20		0.45	2.94	**0.022**	**0.78**

Comparisons between two tasks or conditions were performed across five temporal bins (PreStim, Post1, Post2, Post3, and Post4). Significant results are highlighted in bold.

#### ALPHA BAND (Figure 3C, lower row)

The average time course of the alpha frequency range in the Rfr ROI showed, in general, an increase in power after the stimulus presentation (Table 1). However, only the learning tasks (VSEQ1 and VSEQ5), but not VRAN, showed a significant effect of temporal intervals (Table 2). Similar results were obtained for alpha power in the ROP ROI. In the RFr, direct comparison of VSEQ1 and VRAN showed higher increase of alpha power in VSEQ1 (Table 3), which was more evident just after the stimulus appearance (POST1, p = 0.0005). No significant differences were found between VSEQ5 and VSEQ1 as well as between VSEQ5 and VRAN. In the ROP ROI, alpha power was in general lower in VSEQ1 compared to VRAN (Table 3), with significant decrements in POST 3 (500–750 ms, p = 0.01) and POST4 (750–1000 ms, p = 0.0034). Similar trends were found for the difference between VRAN and VSEQ5 (post-hoc test: both intervals: p = 0.02; p = 0.02).

In summary, we found that sequence learning in the early stages (VSEQ1 vs VRAN) was characterized by decreased theta power in ROP in the pre-stimulus window, followed by increased alpha power in RFr in POST1; increased theta power in AFr in POST2, and decreased alpha power in ROP in both POST3 and POST4, which was still significant in the later stages of learning (VSEQ5 vs. VRAN).

#### Learning-related spectral changes correlate with behavioral indices

**Figure 4:**
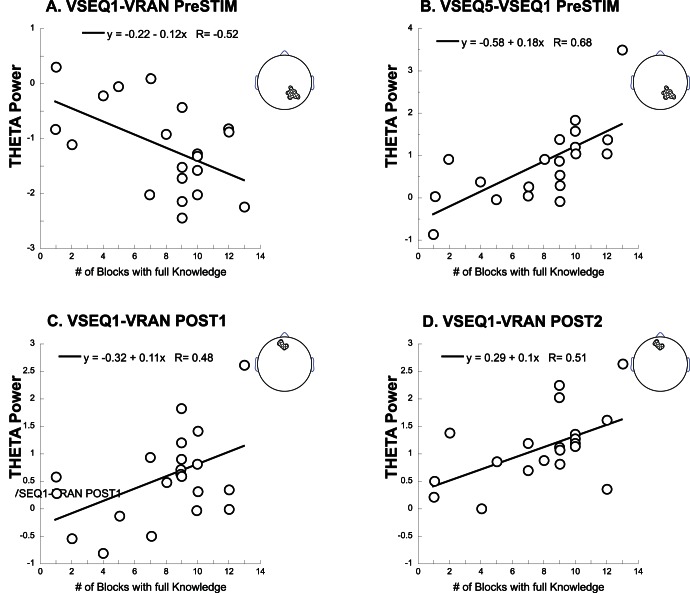
Correlation results. Scatter plots of the correlation between the number of blocks with full sequence order knowledge (i.e., learning indices) and changes in theta power in ROP (top, A. and B.) and AFr (bottom, C. and D.) during learning for time windows where significant power changes occurred. The five 250 ms temporal windows are defined in figure 3.

We next computed the correlation between the general learning index (i.e., number of blocks with full sequence order knowledge) and the significant EEG changes occurring during the early stage of learning (VSEQ1-VRAN) across subjects. We found significant correlations only for theta-related activity changes (Figure 4). Specifically, higher learning indices in the early sequence exposure were associated with greater decrease of theta power in the ROP region in the pre-stimulus window (r = −0.52, p<0.05, Figure 4A) and greater theta increase in the AFr region during pre-stimulus, POST1 and POST2 (r>0.48, p<0.05, Figure 4C & D). Finally, higher learning indices were associated with greater increase of theta in the ROP region in the late phase of learning (VSEQ5-VSEQ1) during the pre-stimulus interval (r = 0.68, p<0.05, Figure 4B).

Altogether, these results suggest that theta activity in the AFr and ROP regions plays a major role in the learning of the sequence order.

### Learning Leaves a Local Trace in the Resting State Spontaneous EEG

**Figure 5:**
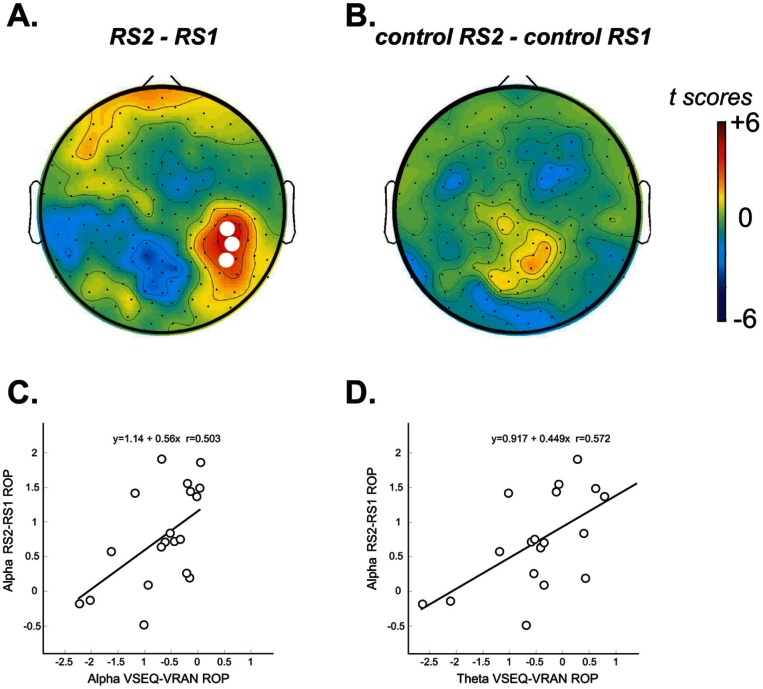
Resting state naTop row: Changes in spontaneous resting state EEG after forty-minute sequence learning tasks (A.) and after forty-minute performance in a variety of different motor tasks (B.). White circles indicate electrodes with significant power differences between RS2 and RS1. Bottom Row: Correlations between the changes in resting state EEG (alpha range) and the VSEQ-VRAN changes observed 250 ms pre- and post-stimulus in the alpha (C) and theta ranges (D).

To determine whether the activity occurring during VSEQ left a lasting trace after the learning session, we compared the resting state EEG during eyes open before and after VSEQ. As shown in figure 5, we found a significant increase of alpha power in the electrodes over a right occipito-parietal area, partially overlapping with ROP, the area showing spectral changes during the task. The mean of alpha power averaged over the significant electrodes was −0.04 dB (S.E. 0.31) for RS1 and 0.69 dB (S.E. 0.24) for RS2. Importantly, no significant changes were observed in the control experiment (*control* RS2 vs *control* RS1). We then correlated the changes in the resting state EEG (RS2-RS1, averages over the significant electrodes, see Figure 5) with the significant EEG changes observed during learning in ROP area for both theta and alpha ranges. The changes in the resting state EEG were positively correlated with the VSEQ-VRAN changes observed 250 ms pre- and post-stimulus in the alpha (r = 0.57, p<0.01) and theta ranges (r = 0.50, p<0.01; see Figure 5 C and D). These correlations were significant also when comparing separately VSEQ1-VRAN (alpha: r = 0.49, p<0.01; theta: r = 0.48, p<0.01) and VSEQ5-VRAN (alpha: r = 0.55, p<0.01; theta: r = 0.50, p<0.01) changes. Finally, no significant correlations were found between the changes in the resting state EEG and learning indices.

## Discussion

This is the first study to characterize theta and alpha changes during the declarative learning of a visual sequence order and to identify post-task local changes in the resting-state EEG over an area active during the task. Specifically, during the task, electrodes over frontal and occipito-parietal regions showed learning-related changes in both alpha and theta power, which were temporally linked to the stimulus appearance. This provides the first EEG evidence of involvement of alpha and theta changes in the formation of declarative memory in a visuo-spatial task that requires acquisition, retrieval and manipulation of information in working memory. Moreover, after the task, spontaneous EEG recordings showed a selective increase of alpha power in electrodes over a right occipito-parietal area, partially overlapping with those active during the task. The post-task changes at rest correlated with the EEG changes during the task, suggesting that they may well represent a trace of use-dependent plasticity.

### Alpha and Theta Oscillatory Activity Changes During Declarative Spatial Learning

Learning during VSEQ involves many processes: initially, attending stimulus appearance and directing visual attention toward the target (processes that are in common with VRAN), processing and encoding it; later on, retrieving, predicting and checking successive target appearance. In summary, visuo-spatial attention, working memory, controlling mechanisms, encoding and retrieval are needed at various points during each target occurrence, in different degrees depending upon the learning stage. The global pattern of activation reflecting these processes has been described with O^15^-PET and involves right visual association cortices, right pre-SMA, dorsolateral prefrontal cortices bilaterally, anterior cingulate, SMA and cerebellum [9], [12], [13]. Although scalp-recorded EEG has low spatial resolution, the topography of alpha and theta power changes in this study was similar, with a right-hemisphere lateralization in electrodes over ROP, RFr and AFr. The spectral changes over ROP electrodes probably represent activity of visual associative areas (reflecting spatial-attentive processes); those over RFr and AFr electrodes might represent activity of frontal regions, including the anterior cingulate (reflecting working memory, attention, controlling and encoding).

The high temporal resolution of EEG allowed us to analyze the spectral changes relatively to the stimulus occurrence. In VRAN, VSEQ1 and VSEQ5, stimulus appearance induced a power increase, lasting about 500 ms, more evident in theta (Figure 3). On average, during VSEQ compared to VRAN, alpha and theta power was lower over ROP electrodes, while the reverse was true for electrodes over frontal areas. As target characteristics and presentation were the same in all tasks, the differences between VRAN and VSEQ must reflect learning-related processes.

Theta power increase (synchronization) in scalp-recorded EEG is considered a hallmark of cortico-hippocampal interplay at the cortical end, representing encoding of new information [32], [33], [34]. Accordingly, in VSEQ1 -where encoding was greatest- compared to VRAN, we found a general theta increase over AFr electrodes that reached significance 250–500 ms post-stimulus. This might represent anterior cingulate activity [35], an area anatomically linked to the hippocampus and other subcortical structures. Conversely, in VSEQ1, theta power was significantly lower in ROP electrodes, just before stimulus appearance. Theta decreases had been interpreted as activity aimed at inhibiting task-irrelevant information or interfering processes, a phenomenon described in a few cases [36]. In VSEQ1, suppression of irrelevant activity in occipito-parietal areas before the temporally expected stimulus appearance would prepare for the acquisition of the incoming target, as these regions are specifically involved in spatial attention and spatial working memory [9], [37]. Finally, the positive correlation between learning rates and theta-related changes in AFr and ROP confirms that theta activity may be associated with efficient encoding of new spatial information.

Alpha power was generally lower in VSEQ compared to VRAN over ROP electrodes, mostly in the last 500 ms post-stimulus. Alpha power decreases have been associated with retrieval of information from memory that is used for encoding, a sort of memory reactivation for further manipulation [38], [39]. The topographical and temporal patterns of the changes suggest that this might be indeed the case. As discussed previously, ROP is involved in spatial working memory processes and the nature of our task, beyond stimulus processing, requires linking a target position with that of other targets in the sequence by accessing and retrieving previously acquired information. The fact that these changes were still significant in VSEQ5, when acquisition was minimal and sequence knowledge complete, confirms previous conclusions of semantic learning tasks that alpha decreases indeed represent processes related to recognition and retrieval through access to previously stored information [40], [41], [42]. In summary, these alpha changes may not merely reflect attentional processes, but may well represent operations intrinsically related to learning. Finally, in VSEQ1 compared to VRAN, we observed an increase in alpha power in RFr, just after stimulus occurrence. Alpha power increases, present in different tasks of working memory and attention, seem to reflect active inhibition of task-irrelevant processes in a sort of top-down control [43], [44], [45]. In particular, Sauseng and colleagues [45] demonstrated stronger pre-frontal alpha synchronization and occipital alpha suppression when information was manipulated in working memory compared to tasks of pure retention, with increased functional coupling between prefrontal and occipital sites and alpha latency shifts from prefrontal to occipital sites. Their conclusions that frontal alpha increase represents an active, anterior control over posterior areas during information manipulation are supported by our temporal and topographical patterns in RFr and ROP (figure 3).

In summary, the temporal and topographical patterns of oscillatory activity during VSEQ compared to VRAN suggest that alpha and theta changes are expression of different learning-related processes. We can speculate that, in early learning, the post-stimulus alpha power increase in RFr and the successive alpha power decrease in ROP might reflect working memory-related manipulations, in which new information is embedded in retrieved memory. When acquisition is completed (VSEQ5), retrieval of previously stored information occurs in the context of sequence recognition. The pre-stimulus ROP theta decrease might work as a pre-emptive strike to prepare for encoding of new material, while AFr theta increase 250 ms post-stimulus might represent encoding of new information, explaining why is evident in VSEQ1 and not in VSEQ5. Finally, the role of theta activity in the efficient encoding of new spatial information is also supported by its correlation with acquisition rates.

### Learning Leaves a Specific Local Trace in the Resting State EEG

After the task, we found local changes in the spontaneous EEG at rest. Indeed, changes in electrophysiological measurements have been described by studies with transcranial magnetic stimulation, after either strenuous or prolonged exercises with and without fatigue [5], [46], [47], [48]. In particular, a recent study [5] has shown that, after ten minutes of uninterrupted finger movements paced by a metronome at 2 Hz, motor cortical excitability decreased; most importantly, this decrease was likely induced by use (i.e., a sign of use-dependent plasticity), as it occurred without signs of neuromuscular fatigue. However, local and task specificity of post-task changes has only been shown in EEG studies: in one, a local and task-specific trace was found in the spontaneous EEG at rest after twenty-four hours of continuous performance [6], [7]; in the other a trace was found after a forty-minute performance [7]. However, in both of them, inferences were made about the locality of the trace with respect to the task, as the changes in the resting state EEG were not directly compared with the EEG activity during the performance. This is the first study that permits to ascertain directly whether the post-task changes in the spontaneous resting-state EEG occurred in the areas involved in the task, as we determined both the temporal evolution of spectral changes during VSEQ and the changes at rest after forty-minute performance.

In the present work, a significant post-task increase of alpha power occurred in electrodes over the right occipito-parietal region overlapping with electrodes that showed significant changes during the task. Importantly, such change was not present after a similar amount of time spent performing a variety of motor tasks that did not encompass sequence learning, suggesting that this local trace was task-specific. The trace magnitude was related, at least partially, to the functional changes occurring in this same area during the tasks, as it significantly correlated with the ROP theta and alpha changes observed during the sequence learning task. As there were no correlations with the learning indices, this trace is likely unrelated to either the level of knowledge achieved or the acquisition rate, but it could rather reflect extensive use of this area during learning. Finally, this trace was frequency-specific, as it occurred in alpha and did not generalize to the entire spectrum.

While alpha increases during tasks likely reflect cortical inhibitory activity aimed at blocking retrieval of interfering responses as previously discussed [38], not much is known about the significance of alpha power increases in resting-state EEG in normal subjects as a consequence of tasks. Changes in alpha power, mostly in terms of decreases, have been described in response to neurological disorders, circadian rhythms, and aging. In a previous work [7], after an implicit visuo-motor adaptation task, we found significant resting-state alpha changes over parietal (decreased power) and frontal (increased power) regions, which were active during the task [11]. We interpreted these findings as traces of the learning-related processes occurred during the forty-minute task. In a following study, we found increases of theta power in areas involved in tasks performed for twenty-four hours. Theta increase at rest has been associated with increase sleep pressure and fatigue [6]. We thus postulate that both alpha and theta increases might represent homeostatic processes following relatively short (alpha) or longer and more intensive (theta) periods of use. It is possible that alpha increases might represent a first step towards long-term potentiation processes to consolidate memory, while the later occurrence of theta might signal off-line activity due to increased neuronal instability and activity-dependent synaptic “overload” [49], [50]. Further studies will address these points and will elucidate the specificity, the time course and the significance of a task’s trace in the spontaneous resting-state EEG.
